# Midwave FTIR-Based Remote Surface Temperature Estimation Using a Deep Convolutional Neural Network in a Dynamic Weather Environment

**DOI:** 10.3390/mi9100495

**Published:** 2018-09-27

**Authors:** Sungho Kim, Jungho Kim, Jinyong Lee, Junmo Ahn

**Affiliations:** 1 Department of Electronic Engineering, Yeungnam University, 280 Daehak-Ro, Gyeongsan, Gyeongbuk 38541, Korea; 2 Agency for Defense Development, 111 Sunam-dong, Daejeon 34186, Korea; jhkim@add.re.kr (J.K.); jinylee@add.re.kr (J.L.); ahnjm@add.re.kr (J.A.)

**Keywords:** midwave infrared, thermal radiation, hyperspectral, remote surface temperature, weather variation, deep learning, regressor, thermal stealth

## Abstract

Remote measurements of thermal radiation are very important for analyzing the solar effect in various environments. This paper presents a novel real-time remote temperature estimation method by applying a deep learning-based regression method to midwave infrared hyperspectral images. A conventional remote temperature estimation using only one channel or multiple channels cannot provide a reliable temperature in dynamic weather environments because of the unknown atmospheric transmissivities. This paper solves the issue (real-time remote temperature measurement with high accuracy) with the proposed surface temperature-deep convolutional neural network (ST-DCNN) and a hyperspectral thermal camera (TELOPS HYPER-CAM MWE). The 27-layer ST-DCNN regressor can learn and predict the underlying temperatures from 75 spectral channels. Midwave infrared hyperspectral image data of a remote object were acquired three times a day (10:00, 13:00, 15:00) for 7 months to consider the dynamic weather variations. The experimental results validate the feasibility of the novel remote temperature estimation method in real-world dynamic environments. In addition, the thermal stealth properties of two types of paint were demonstrated by the proposed ST-DCNN as a real-world application.

## 1. Introduction

The relationships between solar radiance and emitted thermal radiative energy are important for infrared stealth technology. Radiated solar energy (6000 K) heats an object, which then radiates thermal energy according to Planck’s law [1]. One of the core technologies in infrared stealth research is to measure the surface temperature of a remote object.

The surface temperature of an object is independent of the wavelength, and can be estimated from even a single spectral band with a known atmospheric transmissivity and surface emissivity. In addition, the atmospheric conditions are crucial for surface temperature estimation [2]. The atmospheric weather conditions (e.g., temperature, humidity) change dynamically on earth, which leads to wide variations of spectral transmissivity. An incorrect atmospheric transmissivity hinders remote temperature estimation. Many studies have been conducted to measure remote temperature as correctly as possible.

Conventional approaches usually require an atmospheric correction to remotely estimate the temperature [3]. A previous study proposed a temperature estimation using single-channel infrared information and known atmospheric transmissivity [4]. A single band-based temperature emissivity separation (TES) was applied to estimate absolute land surface temperature from the MODerate resolution atmospheric TRANsmission (MODTRAN) -based atmospheric transmissivity information [2]. A single channel-based temperature estimation is impractical because of the inaccurate atmospheric profile information.

A two-channel-based method such as a split window algorithm can estimate the surface temperature as a linear function of two brightness temperatures [5]. Although the split-window method does not require information on the atmospheric transmissivity at the time of the temperature measurement, it requires accurate differential water vapor absorption in two adjacent thermal infrared channels [6].

A four channel-based surface temperature estimation method was proposed in Reference [7]. They reported an improved surface temperature estimation using three longwave thermal channels (8.7, 10.8, 12.0 μm) and one midwave thermal channel (3.9 μm) compared to the split window methods.

A multi-channel method was proposed using longwave hyperspectral thermal infrared for high-emissivity surfaces [8]. They used ten manually selected channels and applied a least square minimization to estimate the parameters. The multi-channel method [9] was improved by considering thirty-six channels and unknown emissivity in a linear system [8].

An accurate remote surface temperature estimation is difficult under dynamically varying weather conditions. The above-mentioned approaches (1-channel, 2-channel, 4-channel, and multi-channel) have their own advantages and disadvantages. These methods work if the specific conditions are satisfied, such as the known atmospheric transmissivity, known water vapor contents, and known surface emissivity. On the other hand, they cannot guarantee the temperature accuracy if the required atmospheric information is unavailable online or the weather conditions change abruptly.

In this paper, the problem of a remote surface temperature estimation in a dynamic weather environment is solved by focusing on the sensor, database (DB), and deep learning scheme. A new hyperspectral thermal infrared camera (HYPER-CAM MWE, TELOPS, Quebec, QC, Canada) was adopted to analyze both the solar radiance and thermal radiation in the 1.5–5.5 μm band. This hyperspectral camera can provide 374 spectral bands with a calibrated spectral radiance. A dynamic weather database was recorded three times a day (10:00, 13:00, and 15:00) for 7 months to cover a wide range of atmospheric variations in a coastal environment. The proposed surface temperature-deep convolutional neural network (ST-DCNN) can estimate the temperatures by learning the network on a huge spectral DB.

The remainder of this paper is organized as follows. Section 2 introduces the background of Fourier transform infrared (FTIR)-based brightness temperature measurement process. Section 3 explains the overall structure of the paper, including the hyperspectral database construction method and deep convolutional neural network-based temperature estimation with the ST-DCNN. Section 4 evaluates the remote surface temperature estimation performance of the proposed method by comparing it with the baseline methods. The paper is concluded in Section 5.

## 2. Background: Passive Open Path Fourier Transform Infrared (OP-FTIR)

**Figure 1:**
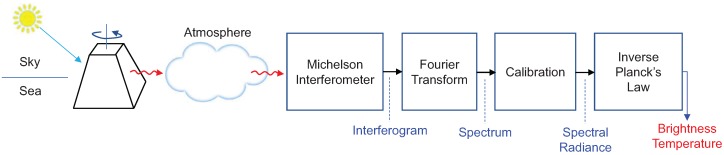
Overall process of the brightness temperature extraction using the passive open path Fourier transform infrared (FTIR) imaging system.

The remote surface temperature estimation was based on the brightness temperature as shown in Figure 1. The research objective was to estimate the temperature of a surface heated by solar energy. The surface heated by direct sunlight was rotated $180^{\circ }$ after 20 min to determine the thermal radiation. The thermal energy radiated by the rotated surface passes through the atmosphere, which alters the spectral radiation. The atmospheric transmittance changes according to the molecular contents, such as carbon dioxide and water vapor. Additional thermal energy coming from the atmosphere was added to the remote surface radiation. This thermal radiation was recorded in a Michelson interferometer and the spectra were obtained by applying the Fourier transform to the interferograms. The brightness temperatures were obtained through the spectral-radiometric calibration and inverse of Planck’s law.

### 2.1. Passive Open Path Michelson Interferometer

**Figure 2:**
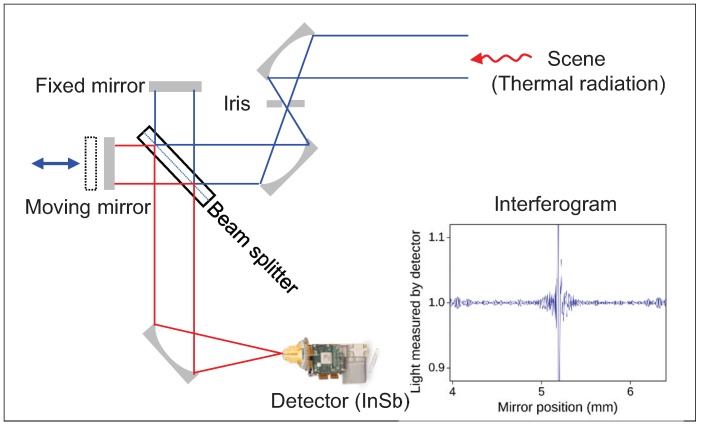
Basic diagram of a Michelson interferometer for passive open path applications.

An open path Michelson interferometer can be prepared using an active or passive approach for outdoor applications [10]. The former uses an active IR source that usually heats between 1000 and 1800 ${}^{\circ }$C. The maximum range for an active source open path Fourier transform infrared (OP-FTIR) system is approximately 500 m. On the other hand, the latter has no sending unit. The infrared source is generally the sun (absorption) or a preheated object surface (emission). In the sun source, solar energy passes through gaseous material and the amount of absorbed energy can be measured. Figure 2 presents the basic diagram of a passive open path Michelson interferometer. Although the sensitivity of the passive OP-FTIR is generally less than that of the active method, the effective range is longer (i.e., up to several kilometers), which is suitable for remote sensing applications.

**Figure 3:**
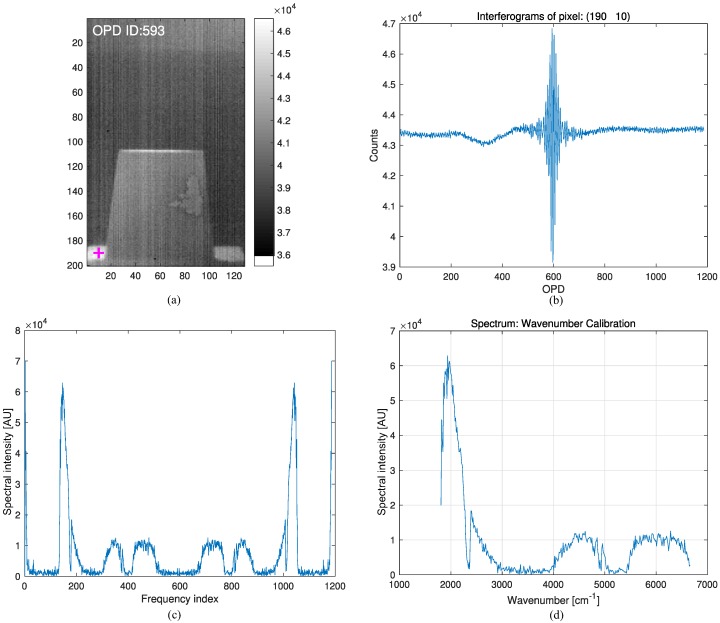
Spectral calibration process: (**a**) interferogram image at optical path difference (OPD) ID = 592 (ZPD); (**b**) interferogram at a pixel ((row, col) = (190, 10)); (**c**) fast Fourier transform (FFT) results; (**d**) wavenumber calibration results.

A Michelson interferometer receives an input beam of radiation, divides the beam into two paths, and then recombines the two beams after a path difference [11]. A beam splitter divides the beam into two paths, and two mirrors, where one of the mirrors is movable, can make a path difference after the beams reflect off the mirrors. The recombined two beams at the beam splitter generate an interference pattern that is recorded in the detector (indium antimonide, InSb). This interference pattern is called an interferogram, which is the raw data from a passive OP-FTIR sensor. Figure 3a presents an interferogram image at zero path difference (ZPD), whose equivalent optical path difference (OPD) ID is 593. Figure 3b gives an example of the whole interferogram at pixel (190, 10). The following subsections outline the mathematical formulations of how to convert raw interferograms to a brightness temperature [12].

### 2.2. Fourier Transform

The intensity as a function of the path difference in the interferometer *p* and wavenumber $\tilde{\nu }=1/\lambda$ is expressed as Equation (1) [13]:

(1) $$\begin{array}{c} I\left(p,\tilde{\nu }\right)=I\left(\tilde{\nu }\right)\left[1+cos\left(2\pi \tilde{\nu }p\right)\right], \end{array}$$

where $I(\tilde{\nu })$ is the spectrum to be found. The total intensity at the detector is

(2) $$\begin{array}{c} I\left(p\right)=\int_{0}^{\infty }I\left(p,\tilde{\nu }\right)d\tilde{\nu }=\int_{0}^{\infty }I\left(\tilde{\nu }\right)\left[1+cos\left(2\pi \tilde{\nu }p\right)\right]d\tilde{\nu }. \end{array}$$

This is a Fourier cosine transformation. The inverse transform can extract the desired spectrum ($I(\tilde{\nu })$) using Equation (3). The fast Fourier transform (FFT) is used in the implementation stage. Figure 3c shows the results of spectrum extraction by applying the FFT to the interferogram (Figure 3b). The unit of the y-axis in Figure 3c is just spectral intensity in arbitrary units.

(3) $$\begin{array}{c} I\left(\tilde{\nu }\right)=4\int_{0}^{\infty }\left[I\left(p\right)-0.5I\left(p=0\right)\right]cos\left(2\pi \tilde{\nu }p\right)dp. \end{array}$$

### 2.3. Spectral Wavenumber Calibration

A spectral or wavenumber calibration of the FTIR can be performed using the sampling theorem in digital signal processing [14]. The HYPER-CAM MWE uses a HeNe laser with wavelength λ=632.8 nm, whose light also travels through the interferometer. The peaks of the laser signal are used to sample the received infrared (IR) source. The Nyquist sampling rate (${\tilde{\nu }}_{Nyquist}$) was $1/\lambda \cdot 0.01\;({\mathrm{cm}}^{-1})$ and the wavenumber spacing ($\Delta \tilde{\nu }$) was calculated by ${\tilde{\nu }}_{Nyquist}/N$, where the number of samples is denoted as *N*. In the present system, *N* was 1186 and $\Delta \tilde{\nu }$ was $13.3244\;\mathrm{cycles}\;({\mathrm{cm}}^{-1})$. Therefore, the ideal wavenumber range was 0 to $13.3244\times 1186/2=7901.4\;({\mathrm{cm}}^{-1})$. On the other hand, the response bands of the detector (InSb) were 1807.9–$6659.3\;({\mathrm{cm}}^{-1})$. Only 374 wavenumbers were used in the present FTIR system. Figure 3d shows the wavenumber calibrated spectrum.

### 2.4. Radiometric Calibration

A radiometric calibration is needed to acquire calibrated spectra in units of radiance [15]. In a space application, a blackbody (BB) and cold space can be used [16]. The HYPER-CAM MWE can provide the spectral radiance data using two BBs (hot, cold) [16,17].

**Figure 4:**
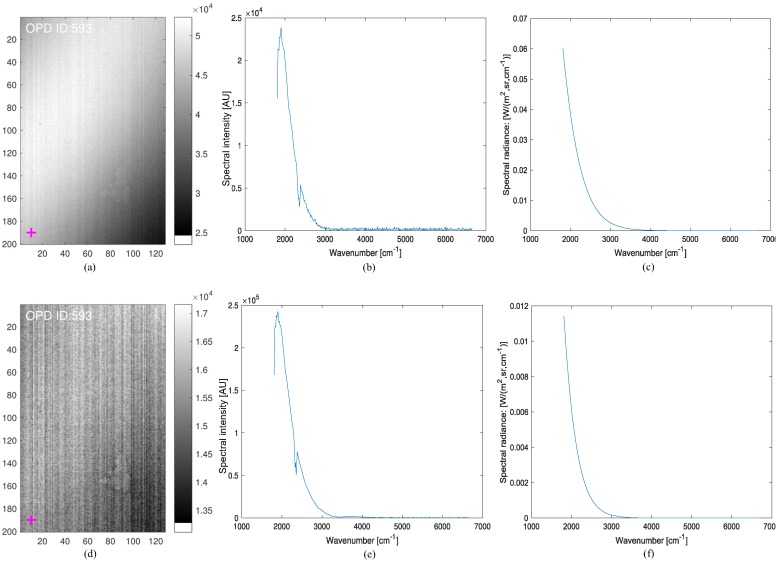
Blackbody spectrum and spectral radiance extraction for a radiometric calibration: (**a**) interferogram image of a hot blackbody ($95{\;}^{\circ }$C); (**b**) spectrum of a hot blackbody; (**c**) calculated spectral radiance at hot temperature; (**d**) interferogram image of a cold blackbody ($25{\;}^{\circ }$C); (**e**) spectrum of a cold blackbody; (**f**) calculated spectral radiance at cold temperature.

**Figure 5:**
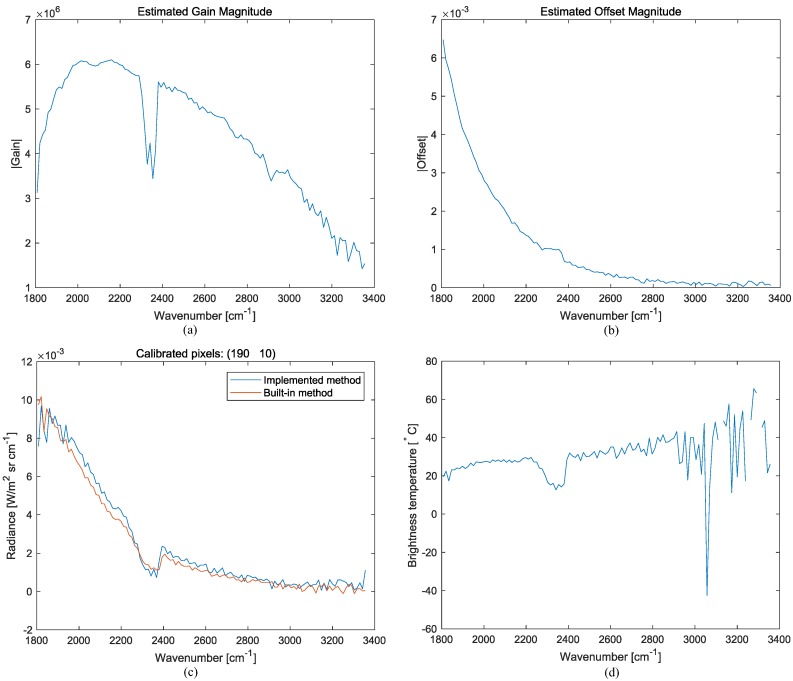
Radiometric calibration and brightness temperature extraction: (**a**) estimated gain magnitude; (**b**) estimated offset magnitude; (**c**) comparison of the radiometric calibration between the implemented method and built-in method; (**d**) calculated brightness temperature.

The radiometric calibration involves characterizing the FTIR response by a linear equation, as expressed in Equation (4):

(4) $$\begin{array}{c} M\left(\tilde{\nu }\right)=G\left(\tilde{\nu }\right)\cdot \left(L\left(\tilde{\nu }\right)+O\left(\tilde{\nu }\right)\right), \end{array}$$

where $M(\tilde{\nu })$ is the complex spectrum from the instrument measurement, and $G(\tilde{\nu })$ and $O(\tilde{\nu })$ are the gain and offset of the instrument, respectively. $L(\tilde{\nu })$ means the true spectral radiance $({W/(m}^{2}\cdot \mathrm{sr}\cdot {\mathrm{cm}}^{-1}))$. The gain and offset can be estimated by measuring the radiance of two known BBs. The theoretical radiance follows Planck’s law, defined in Equation (5):

(5) $$\begin{array}{c} L_{BB}\left(\tilde{\nu },T\right)=\frac{2hc^{2}{\tilde{\nu }}^{3}}{e^{hc\tilde{\nu }/kT}-1}, \end{array}$$

where $L_{BB}\left(\tilde{\nu },T\right)$ denotes the spectral radiance (${W/(m}^{2}\cdot \mathrm{sr}\cdot {\mathrm{cm}}^{-1})$) of a blackbody, *h* is Planck’s constant, *c* is the speed of light, *k* is Boltzmann’s constant, and *T* is the blackbody temperature (*K*). If two BBs with known temperatures ($T_{H},T_{C}$) are given, two radiances ($L\left(\tilde{\nu },T_{H}\right),L\left(\tilde{\nu },T_{C}\right)$) and two corresponding spectral measurements ($M_{H}\left(\tilde{\nu }\right),M_{L}\left(\tilde{\nu }\right)$) are prepared. The unknown gain and offset can be obtained by solving the two equations: Equations (6) and (7). Figure 4 shows the acquired interferograms, spectra (arbitrary units), and calculated spectral radiances for the hot ($95{\;}^{\circ }$C) and cold ($25{\;}^{\circ }$C) blackbodies (first row and second row, respectively). Figure 5a,b show the estimated gain magnitude and offset magnitude at pixel (190, 10), respectively. Figure 5c presents the final estimated spectral radiance at the same pixel by comparing the data obtained by the built-in (TELOPS) calibration method. The built-in method means the spectral and radiometric calibration provided by the TELOPS instrument (Quebec, QC, Canada), which is regarded as truth. Note that similar spectral radiance can be obtained in the spectral range of 1807.9–3355.7 (${\mathrm{cm}}^{-1}$) using Equation (8).

(6) $$\begin{array}{c} G\left(\tilde{\nu }\right)=\frac{M_{H}\left(\tilde{\nu }\right)-M_{C}\left(\tilde{\nu }\right)}{L\left(\tilde{\nu },T_{H}\right)-L\left(\tilde{\nu },T_{C}\right)} \end{array}$$

(7) $$\begin{array}{c} O\left(\tilde{\nu }\right)=\frac{M_{C}\left(\tilde{\nu }\right)L\left(\tilde{\nu },T_{H}\right)-M_{H}\left(\tilde{\nu }\right)L\left(\tilde{\nu },T_{C}\right)}{M_{H}\left(\tilde{\nu }\right)-M_{C}\left(\tilde{\nu }\right)} \end{array}$$

If an unknown scene spectrum is measured ($M_{S}\left(\tilde{\nu }\right)$), the radiometrically calibrated radiance ($L_{S}\left(\tilde{\nu }\right)$) can be obtained using Equation (8):

(8) $$\begin{array}{c} L_{S}\left(\tilde{\nu }\right)=\frac{L\left(\tilde{\nu },T_{H}\right)-L\left(\tilde{\nu },T_{C}\right)}{M_{H}\left(\tilde{\nu }\right)-M_{C}\left(\tilde{\nu }\right)}\cdot M_{S}\left(\tilde{\nu }\right)-\frac{M_{C}\left(\tilde{\nu }\right)L\left(\tilde{\nu },T_{H}\right)-M_{H}\left(\tilde{\nu }\right)L\left(\tilde{\nu },T_{C}\right)}{M_{H}\left(\tilde{\nu }\right)-M_{C}\left(\tilde{\nu }\right)}. \end{array}$$

### 2.5. Brightness Temperature

The amount of spectral radiance energy can be converted into equivalent brightness temperatures [1]. By inverting Equation (5), the temperature T(K) can be obtained as

(9) $$\begin{array}{c} T=\frac{\left(hc/k\right)\tilde{\nu }}{ln[2hc^{2}{\tilde{\nu }}^{3}/L_{S}\left(\tilde{\nu }\right)+1]}. \end{array}$$

Figure 5d shows the spectral brightness temperature by applying Equation (9) to the calibrated spectral radiance at pixel (190, 10). Note that there were inaccurate values due to the spectral radiance noise. The gaps in Figure 5d were generated by negative spectral radiances that were obtained during the radiometric calibration for noisy spectral radiance values. Negative spectral radiances cannot provide physical temperatures in Equation (9).

## 3. Proposed Temperature Estimation: ST-DCNN

**Figure 6:**
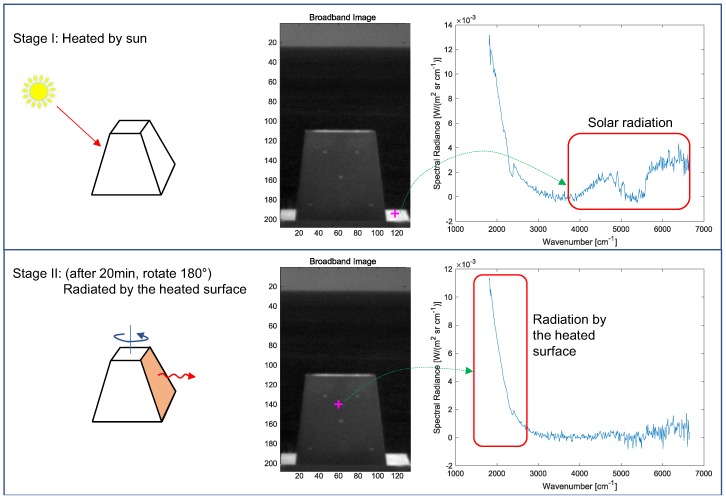
Thermal stealth evaluation flow caused by solar radiation at day time.

An evaluation of thermal infrared stealth property is important for various applications, such as ships, cars, and houses. This paper focuses on the effects of solar radiation on objects. As shown in Figure 6 (top row), the sun heats up the surface of an object painted with specially developed materials. After 20 min (thermal equilibrium), the heated surface is rotated by $180^{\circ }$ and radiates thermal energy (Figure 6, bottom row), which is measured by an FTIR detector. Note that the solar radiation (6000 K) is dominant in the higher wavenumber band (short wavelength region: 1.5–3.0 μm, or 3333–6667 cm${}^{-1}$) and the energy is used to heat the surface (approximately 0–40${}^{\circ }$) of an object as indicated by the cross point. Therefore, the radiation by the heated surface is dominant in the lower wavenumber band (mid-wavelength region: 3.6–5.5 μm, or 1818–2778 cm${}^{-1}$). The strength of the solar radiation in the shadowed region (opposite side of the direct sun) decreased to 10%–20%.

**Figure 7:**
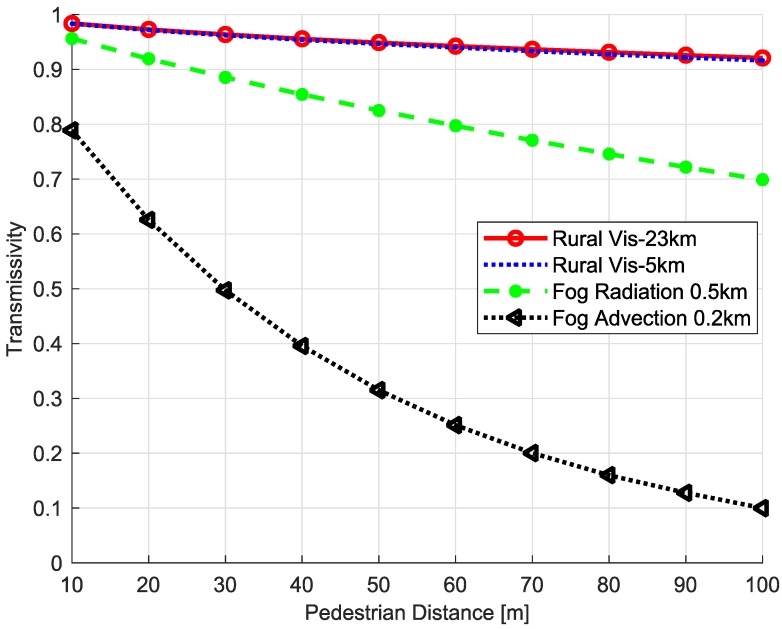
Atmospheric transmissivity variations according to the distance and weather conditions.

In remote surface temperature sensing, the infrared spectral radiance at the FTIR detector can be represented by radiative transfer, as per Equation (10):

(10) $$\begin{array}{c} L\left(\tilde{\nu }\right)=\varepsilon_{0}\left(\tilde{\nu }\right)L_{BB}\left(\tilde{\nu },T\right)\tau \left(\tilde{\nu }\right)+L_{a}\left(\tilde{\nu }\right), \end{array}$$

where $\varepsilon_{0}$ is the surface spectral emissivity, $\tau (\tilde{\nu })$ is the transmissivity at the FTIR detector, and $L_{a}\left(\tau \left(\tilde{\nu }\right)\right)$ represents the thermal path radiance [18]. The surface temperature (*T*) can be estimated from the spectral radiance ($L(\tilde{\nu })$) if the object emissivity, atmospheric transmissivity, and path radiance are available. On the other hand, the environmental parameters are difficult to estimate in real-time due to the wide variations of weather conditions. Figure 7 gives an example of atmospheric transmissivity according to the object distance and weather conditions. Therefore, the one-channel method (atmospheric transmissivity is required), two-channel method (water vapor content is required), and multi-channel method (surface emissivity is required) are not applicable.

The key ideas are based on three aspects. First, a midwave thermal hyperspectral imager (HYPER-CAM MWE) is adopted to extract the spectral information. The midwave thermal hyperspectral images usually show lower sensitivity than longwave thermal hyperspectral images on the surface emissivity in a remote temperature estimation [19]. The surface emissivity can affect the temperature estimation. The low sensitivity means that the uncertainty of emissivity produces low uncertainty in temperature estimation. On a normal surface, the typical values of emissivity for MWIR are approximately 0.90–0.95. Second, the imager can acquire huge hyperspectral radiance and a temperature database at various times (10:00, 13:00, 15:00), seasons (winter, spring, summer), and weather conditions (clear, cloudy, foggy). Third, a novel surface temperature-deep convolutional neural network (ST-DCNN) was adopted to estimate the remote surface temperatures by learning the network with huge spectral radiance DB (equivalently, brightness temperature DB).

**Figure 8:**
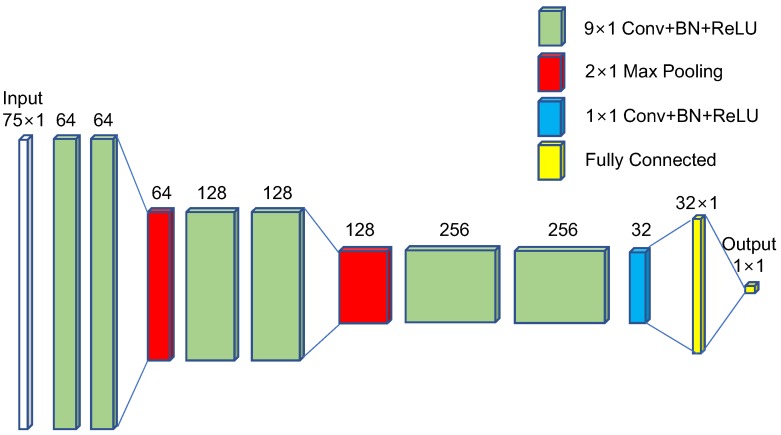
Proposed structure of the surface temperature-deep convolutional neural network (ST-DCNN).

The proposed ST-DCNN consisted of 27 layers, as shown in Figure 8. The input data size was 75×1, corresponding to the midwave band (1807–2270 cm${}^{-1}$ or 3.6–5.5 μm). Two 9×1 convolutions with 64 filters, batch normalization (BN), and rectified linear unit (ReLU) were conducted to extract the temperature features in the spectral domain. The 2×1 max pooling can reduce the dimensions by removing the redundant features. Such processes (except for the last layer-1×1 Conv + BN + ReLU) were repeated twice to extract the higher spectral temperature. A 1×1 convolution was used to reduce the channel size with the same spectral feature size. The last two fully connected layers were used to regress the physical surface temperature. L2 norm was used to calculate the loss.

## 4. Experimental Results

**Figure 9:**
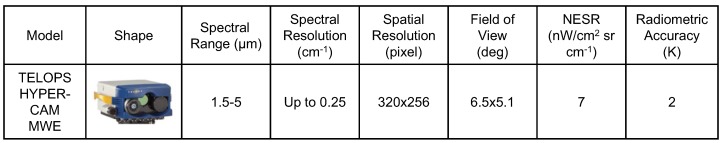
Specifications of the TELOPS Hyper-Cam MWE sensor.

The TELOPS HYPER-CAM MWE model was used in this study, as shown in Figure 9. This model can provide a high-resolution spectrum by a Michelson interferometer (TELOPS, Quebec, QC, Canada) from the midwave to the shortwave band. The noise equivalent spectral radiance (NESR) is 7 $({\mathrm{nW}/(\mathrm{cm}}^{2}\cdot \mathrm{sr}\cdot {\mathrm{cm}}^{-1}))$ and the radiometric accuracy is approximately 2 K.

The object surface was painted with a gray color and was located in a coastal area with a 78 m distance to the FTIR sensor system. The object surface data were acquired from 1 December–30 June, three times a day (10:00, 13:00, 15:00) with 75 spectral bands (3.6–5.5 μm), as shown in Table 1.

**Figure 10:**
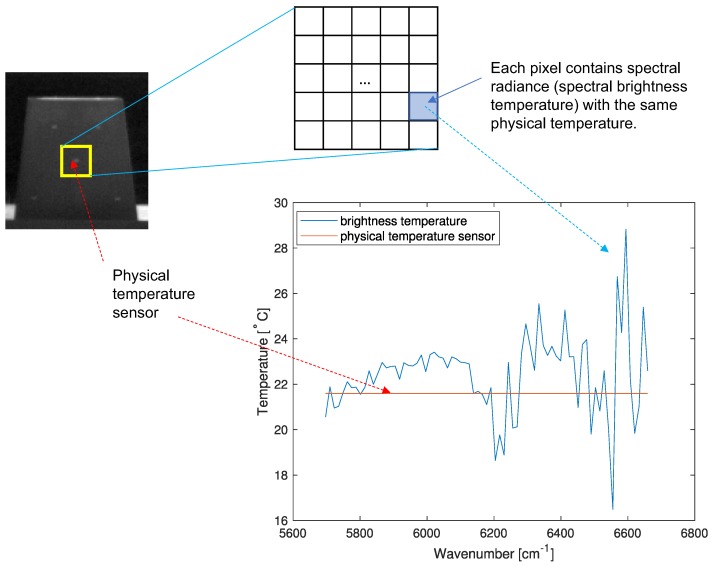
Preparing the spectral brightness temperature and physical temperature pair for deep learning.

Figure 10 presents an example of data preparation for deep learning. The brightness temperature data were extracted at the center region (20×20) and the built-in temperature sensor on the object surface provided the corresponding ground truth temperature information. Therefore, 400 brightness temperature profiles had the same physical temperature.

Table 2 presents details of the spectral brightness temperature for deep learning. The number of valid hyperspectral images was 208, where each image provides 400 spectral brightness temperature profiles. The total number of spectrum–temperature pairs was 82,400, and the DB was divided into three groups: training (80%), validation (10%), and testing (10%).

**Figure 11:**
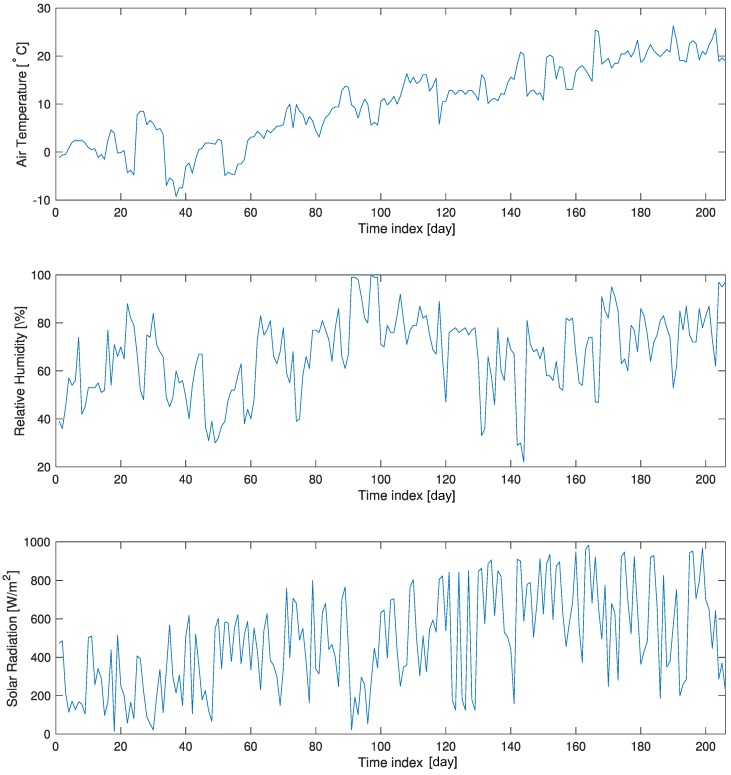
Preparing the spectral brightness temperature and physical temperature pairs for deep learning.

Figure 11 shows the weather variations for the 208 hyperspectral images acquired from 1 December 2017–30 June 2018 in terms of the air temperature, relative humidity, and solar radiation. The air temperature ranged from −10 to $28{\;}^{\circ }$C, the relative humidity varied from 20% to 100%, and the solar radiation varied from 0 to 1000 W/m${}^{2}$ .

The proposed ST-DCNN consisted of 27 layers that were optimized to produce the best regression performance, as shown in Table 3. The number of pooling layers was two, where the max pooling showed better performance than the average pooling. The VGG-style convolutions with the kernel size 9 showed the best root mean square error (RMSE) among the kernel sizes (3, 5, 7, 9, 11, and 13). Additional batch normalization was helpful, and drop-out was useless. A 1×1 convolution was used to reduce the feature dimension, and the regression performance was upgraded if the 1×1 convolution was inserted at the end of the last Conv–BN–ReLU stage. The best performance was seen with 32 fully connected layers, among 8, 16, 32, 64, and 128.

**Figure 12:**
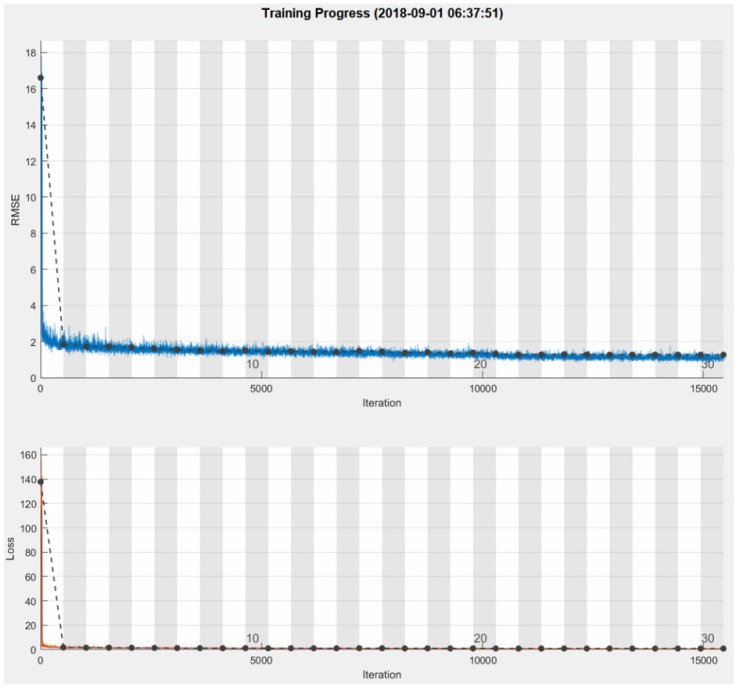
Training process: (**top**) root mean square error curve; (**bottom**) loss curve.

Figure 12 shows the training process with the optimized network parameters. Approximately seven minutes were needed to train the ST-DCNN with a mini-batch size of 128, max epochs of 30, initial learning rate of $1.0\times 10^{-4}$, learning rate drop factor of 0.2, learning rate drop period of 20, and shuffle in every epoch.

**Figure 13:**
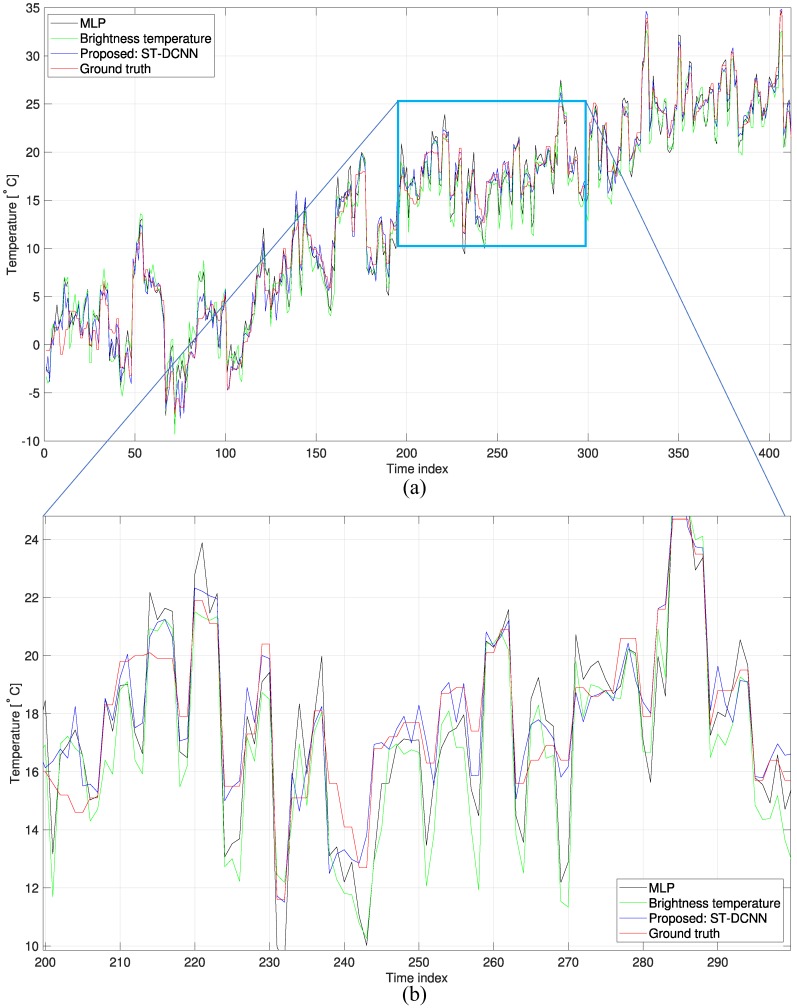
Comparison of the temperature estimation: (**a**) overall temperature; (**b**) enlarged view of the probe region. MLP: multi-layer perceptron.

**Figure 14:**
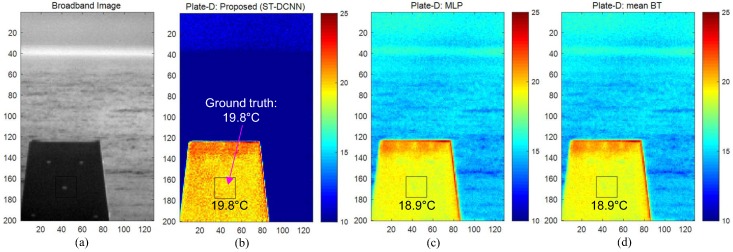
Visualized comparison of temperature estimation: (**a**) broadband image; (**b**) proposed method (ST-DCNN); (**c**) MLP; and (**d**) mean brightness temperature (BT).

The proposed ST-DCNN-based temperature estimation method was compared with the multi-layer perceptron (MLP) [20] and mean brightness temperature [19]. The MLP consisted of two hidden layers with 128 nodes. Figure 13 shows the partial results tested on the unlearned 8240 samples. Figure 13b is an enlarged graph of Figure 13a. Note that the proposed ST-DCNN could predict the true temperatures better than the other methods. Table 4 lists the quantitative comparison results in terms of the RMSE measures. The RMSE value of the direct temperature estimation using the mean brightness temperature was 2.0934 ${}^{\circ }$C and that of the MLP was 1.7863 ${}^{\circ }$C. The proposed ST-DCNN showed an RMSE of 1.1446 ${}^{\circ }$C. This was improved by 45.32% compared to the base method (mean brightness temperature (BT)). Note that the RMSE of the brightness temperature method was similar to the manufacturer’s radiometric accuracy (2 K). Although the RMSE of the proposed ST-DCNN was approximately 1.14 ${}^{\circ }$C, it was reasonably accurate considering the wide weather variations along the three seasons, as shown in Figure 11. Figure 14 presents the remote temperature estimation results for March 29, 15:00 DB. The ground truth temperature of the center region was $19.8{\;}^{\circ }$C, and the proposed ST-DCNN predicted correctly. On the other hand, the MLP and mean BT methods estimated incorrectly by $1{\;}^{\circ }$C.

**Figure 15:**
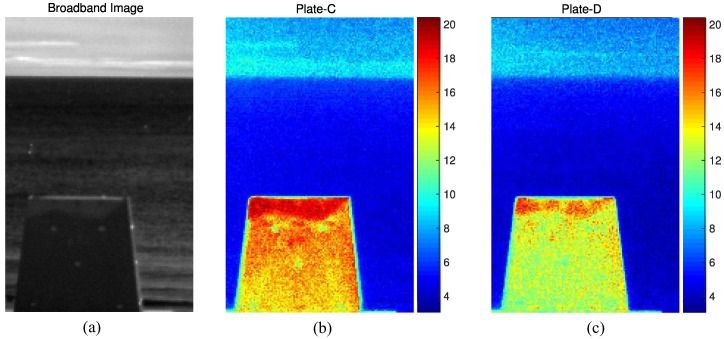
Thermal stealth application of a remote temperature estimation: (**a**) broadband image; (**b**) temperature distribution of Plate-C; (**c**) temperature distribution of Plate-D.

One of many applications of remote temperature estimation is to check the thermal stealth effect of different types of paint on ships, buildings, etc. by solar radiation. Figure 15 shows a quantitative visualization of the physical surface temperature on an object. The surface temperature was estimated using the proposed ST-DCNN with the best network parameters. Figure 15a represents a broadband image of the experimental environment imaged on 5 March 2018. Figure 15b,c show the temperature distributions of Plate-C and Plate-D, respectively. Each plate was painted with different types of surface material. The weather conditions at 15:40 were as follows: atmospheric temperature $10.2{\;}^{\circ }$C, humidity 55%, and solar radiation 550 W/m${}^{2}$. The estimated average temperature of Plate-C was $14.7{\;}^{\circ }$C, whereas that of Plate-D was $12.7{\;}^{\circ }$C. Therefore, the paint on Plate-D had $2.0{\;}^{\circ }$C better thermal stealth capability. In addition, the physical temperature distribution of Plate-C and Plate-D could be compared.

## 5. Conclusions and Further Works

This paper proposed a novel remote surface temperature estimation method using a surface temperature-deep convolutional neural network (ST-DCNN) from midwave hyperspectral thermal images. Estimating the surface temperature remotely in a dynamically varying weather environment is a very challenging problem. The key idea was to apply the specially designed 27-layer deep convolutional neural network to a huge database consisting of spectral radiance–surface temperature sensor pairs. The spectral radiance data were extracted successfully by applying the FFT to the interferogram followed by wavenumber calibration and two blackbody-based radiometric calibrations. The inverse of Planck’s law to the spectral radiance produced the spectral brightness temperature containing both the surface emissivity and atmospheric transmissivity implicitly. The proposed ST-DCNN learned the weight parameters successfully using the 65,920 training samples. According to the experimental results, the proposed ST-DCNN showed an RMSE of $1.1446{\;}^{\circ }$C, which is 45% better than the average brightness temperature. The remote temperature estimation scheme was applied to evaluate the thermal stealth effects by the solar radiance on different types of paint. In the future, a multi-sensor fusion-based deep learning structure will be developed to improve the accuracy of temperature estimation.

## Figures and Tables

**Table 1:** Database acquisition environment of the midwave hyperspectral spectrum.

Object	Location	Duration	Time	Spectral Band
gray painted metal	coast (78 m to sensor)	1/1–6/30	10:00, 13:00, 15:00	3.6–5.5 μm

**Table 2:** Composition of the spectral brightness temperature data for deep learning.

No. of Valid Images	Crop Size	No. of Training (80%)	No. of Validation (10%)	No. of Testing (10%)
208	20×20	65,920	8240	8240

**Table 3:** Optimization results of deep learning parameters. RMSE: root mean square error.

No. of pooling layers	1	**2**	3	4	-
RMSE	1.33	**1.14**	1.26	1.28	-
No. of filters	16–32–64	32–64–128	**64–128–256**	128–256–512	256–512–1024
RMSE	1.29	1.23	**1.14**	1.29	1.37
Filter size	5	7	**9**	11	13
RMSE	1.27	1.22	**1.14**	1.19	1.23
No. of nodes in FC	8	16	**32**	64	128
RMSE	1.19	1.18	**1.14**	1.21	1.23
Dropout rate	0.2	0.3	0.4	0.5	**W/O**
RMSE	1.21	1.21	1.21	1.29	**1.14**
1 × 1 conv. size	16	**32**	64	128	W/O
RMSE	1.23	**1.14**	1.22	1.22	1.25
Batch norm. (BN)	**W/**		W/O		
RMSE	**1.14**		1.77		

**Table 4:** Comparison of the temperature estimation in terms of the RMSE.

Method	RMSE (${}^{\circ }$C)	Improvement (%)
MLP [20]	1.7863	14.67
**Proposed (ST-DCNN)**	**1.1446**	**45.32**
Brightness temperature [19]	2.0934	0
TELOPS MWE: radiometric accuracy	2	-
